# Supplementary material for: Association between oral microbiome and seven types of cancers in East Asian population: a two-sample Mendelian randomization analysis
Source: Front Mol Biosci. 2023 Nov 21;10:1327893. doi: 10.3389/fmolb.2023.1327893 (PMC10702768; doi:10.3389/fmolb.2023.1327893)
Supplement: Supplementary file 1 [file DataSheet1.ZIP › Supplementary Materials/MR plots of saliva/saliva═╝/Prostate cancer/pheno.3098_to_prostate cancer_leave_one_out.pdf]

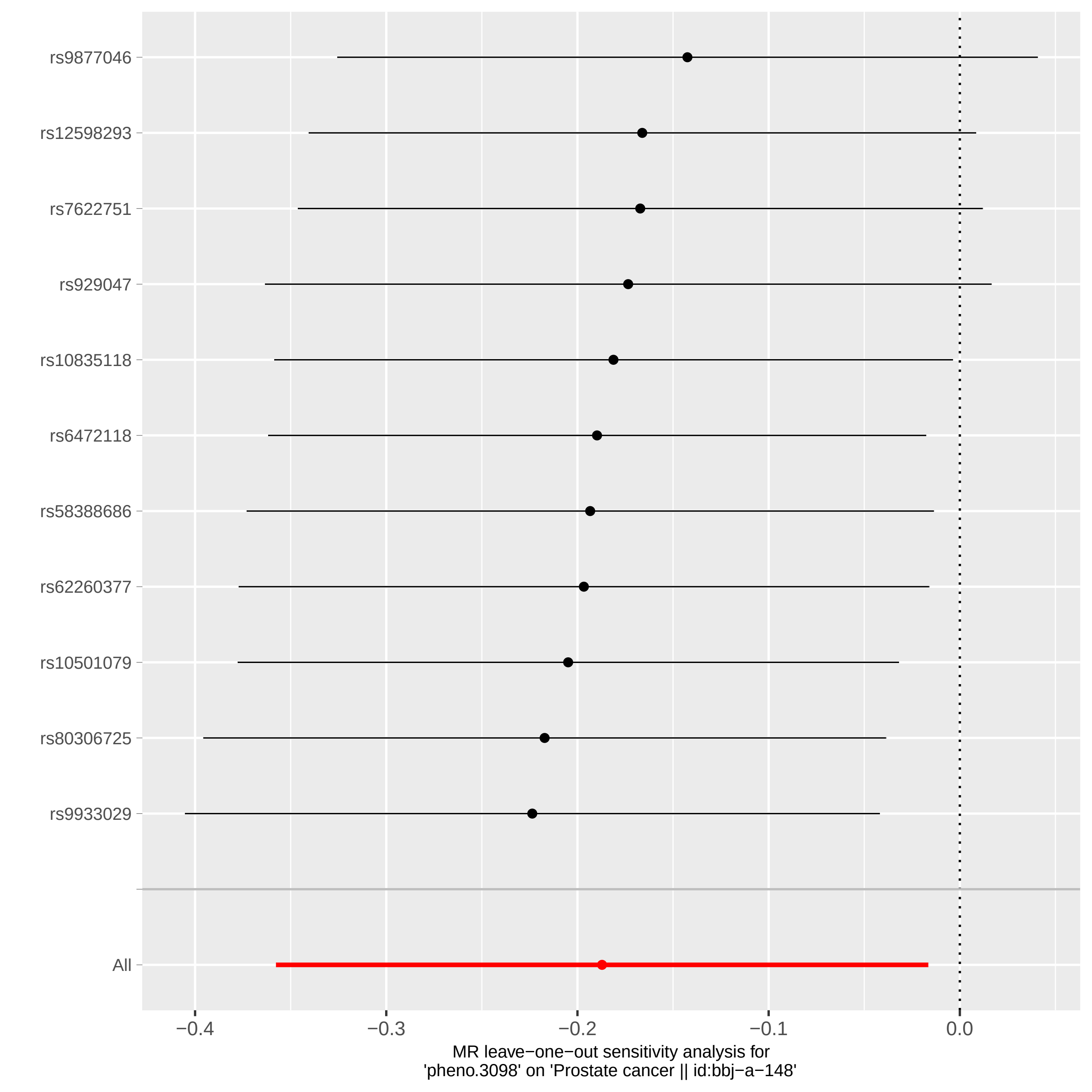

rs9877046
rs12598293
rs7622751
rs929047
rs10835118
rs6472118
rs58388686
rs62260377
rs10501079
rs80306725
rs9933029
All
−0.4
−0.3
−0.2
−0.1
0.0
MR leave−one−out sensitivity analysis for
'pheno.3098' on 'Prostate cancer || id:bbj−a−148'
